# Effect of ferric citrate hydrate on fibroblast growth factor 23 and platelets in non-dialysis-dependent chronic kidney disease and non-chronic kidney disease patients with iron deficiency anemia

**DOI:** 10.1007/s10157-023-02455-6

**Published:** 2024-02-25

**Authors:** Kyoko Ito, Tadao Akizawa, Kojo Arita, Yuko Mitobe, Norio Komatsu

**Affiliations:** 1 Medical Affairs Department, Torii Pharmaceutical Co., Ltd., 3-4-1, Nihonbashi-Honcho, Chuo-Ku, Tokyo, 103-8439 Japan; 2 Doctoral Program in Life Science Innovation (Disease Mechanism), Degree Programs in Comprehensive Human Sciences, Graduate School of Comprehensive Human Sciences, University of Tsukuba, 1-1-1 Tennodai, Tsukuba, Ibaraki 305-8577 Japan; 3 Division of Nephrology, Department of Medicine, Showa University School of Medicine, Namics Shinagawa 301, 4-24-51 Takanawa, Minato-Ku, Tokyo, 108-0074 Japan; 4 Clinical Development Department, Pharmaceutical Division, Japan Tobacco Inc., 3-4-1, Nihonbashi-Honcho, Chuo-Ku, Tokyo, 103-0023 Japan; 5 Department of Hematology, Juntendo University School of Medicine, 2-1-1, Hongo, Bunkyo-Ku, Tokyo, 113-8421 Japan

**Keywords:** Chronic kidney disease (CKD), Ferric citrate hydrate, Fibroblast growth factor 23 (FGF23), Iron deficiency anemia (IDA), Platelet count (PLT)

## Abstract

**Background:**

Iron deficiency anemia (IDA) increases levels of C-terminal fibroblast growth factor 23 (cFGF23) and platelet count (PLT), each of which is associated with cardiovascular events. Therefore, we hypothesized that iron replacement with ferric citrate hydrate (FC) would decrease cFGF23 levels and PLT in patients with IDA.

**Methods:**

In a randomized, open-label, multicenter, 24-week clinical trial, patients with non-dialysis-dependent chronic kidney disease (CKD) and non-CKD complicated by IDA (8.0 ≤ hemoglobin < 11.0 g/dL; and serum ferritin < 50 ng/mL [CKD]; < 12 ng/mL [non-CKD]) were randomized 1:1 to FC-low (500 mg: approximately 120 mg elemental iron/day) or FC-high (1000 mg: approximately 240 mg elemental iron/day). If sufficient iron replacement had been achieved after week 8, further treatment was discontinued.

**Results:**

Seventy-three patients were allocated to FC-low (CKD *n* = 21, non-CKD *n* = 15) and FC-high (CKD *n* = 21, non-CKD *n* = 16). Regardless of CKD status, FC increased serum ferritin and transferrin saturation, did not change intact FGF23 or serum phosphorus, but decreased cFGF23. In FC-low group, median changes in cFGF23 from baseline to week 8 were −58.00 RU/mL in CKD and −725.00 RU/mL in non-CKD; in FC-high group, the median changes were −66.00 RU/mL in CKD and −649.50 RU/mL in non-CKD. By week 8, FC treatment normalized PLT in all patients with high PLT at baseline (>35.2 × 10^4^/µL; FC-low: 1 CKD, 8 non-CKD; FC-high: 3 CKD, 8 non-CKD).

**Conclusion:**

Regardless of CKD status, iron replacement with FC decreased elevated cFGF23 levels and normalized elevated PLT in patients with IDA.

**Clinical trial registration number:**

jRCT2080223943.

**Supplementary Information:**

The online version contains supplementary material available at 10.1007/s10157-023-02455-6.

## Introduction

Iron deficiency anemia (IDA) is the most frequent type of anemia and is highly prevalent among women. Anemia due to iron deficiency is found in 29.6% of non-pregnant women of reproductive age and 36.5% of pregnant women [1]. IDA also frequently complicates chronic heart failure, chronic kidney disease (CKD), and other chronic diseases [2–4]. In addition to the treatment of its underlying cause, oral iron is a first-line therapy for IDA.

Iron deficiency is a risk factor for cardiovascular events and mortality [5, 6], and higher levels of fibroblast growth factor 23 (FGF23) are associated with increased risks of each [7–9]. FGF23 is a peptide hormone that regulates serum phosphorus by promoting renal phosphate excretion and, indirectly, by inhibiting intestinal phosphate absorption. Iron deficiency stimulates production and increases proteolytic cleavage of FGF23, resulting in increases in circulating concentrations of C-terminal cleavage fragments. Therefore, iron deficiency increases levels of FGF23 measured by C-terminal assays (cFGF23) that measure both full-length FGF23 and its C-terminal cleavage fragments, while the full-length FGF23 levels that are detected exclusively by intact FGF23 assays (iFGF23) remain unchanged [10, 11].

Previous studies reported that treatment with intravenous iron such as ferric carboxymaltose (FCM) and iron dextran increased hemoglobin (Hb), serum ferritin, and transferrin saturation (TSAT), and decreased cFGF23 levels in non-CKD patients with IDA. Although there were no significant changes in iFGF23 level in iron dextran-treated patients, FCM increased iFGF23 levels, which led to subsequent decreases in serum phosphorus [12]. No prior studies reported the effect of oral irons on cFGF23 and iFGF23 levels in non-CKD patients with IDA.

IDA can also increase platelet count (PLT), high levels of which are associated with thrombosis [13, 14]. In a previous administrative cohort of patients with IDA, 32.6% had thrombocytosis (PLT > 45 × 10^4^/µL) and 15.8% had thromboses [13]. Administration of an iron-deficient diet induced anemia and thrombocytosis in rats [15], and increased thrombus size in rat models of arterial and venous thrombosis [16]. Administration of oral ferrous succinate decreased PLT below 45 × 10^4^/µL in non-CKD patients with IDA whose PLT were higher than 45 × 10^4^/µL [17]. Therefore, iron replacement with oral iron in non-CKD patients with IDA could be expected to reduce elevated PLT and, perhaps, to reduce risk of thrombosis. No prior studies reported the effect of oral irons on PLT in non-dialysis-dependent (NDD) CKD patients with IDA.

Ferric citrate hydrate (FC, Riona®, 250 mg tablet, Torii Pharmaceutical Co., Ltd., Tokyo Japan) is approved in Japan as an phosphate binder for patients with CKD, including dialysis and NDD [18–20], and as an oral iron for patients with IDA [21, 22]. Ferric citrate (Auryxia®; Akebia Therapeutics, Inc., Cambridge, MA, USA), which has the same active ingredient as Riona®, is approved in the USA to treat hyperphosphatemia in CKD patients with dialysis, and IDA in patients with NDD-CKD [23, 24].

We previously reported the efficacy and safety of FC as iron replacement therapy in Japanese patients with IDA [22]. In the present study, we conducted a post-hoc analysis of the effects of FC on iFGF23, cFGF23, and PLT in addition to iron- and erythrocyte-related parameters.

## Materials and methods

### Study design

We conducted a randomized, open-label, multicenter, 24-week trial at 31 centers in Japan from July 2018 to December 2019 (jRCT2080223943) to investigate the effects of iron replacement with FC in Japanese patients with IDA [22].

### Study population

Key inclusion criteria were Japanese patients aged ≥20 years without and with CKD (defined as estimated glomerular filtration rate based on serum creatinine <60 mL/min/1.73 m^2^) whose Hb levels were 8.0 ≤ Hb < 11.0 g/dL and who met CKD-specific criteria for serum ferritin: <50 and <12.0 ng/mL in non-CKD patients.

Key exclusion criteria were anemia due to conditions other than iron deficiency; serum phosphorus level <2.5 mg/dL or ≥4.5 mg/dL; planned initiation of maintenance dialysis or kidney transplantation during the study period; history of current or previous malignancy within the past 5 years; treatment with iron-free medications for hyperphosphatemia within the previous 2 weeks; treatment with oral iron, intravenous iron, or iron-containing medications for hyperphosphatemia within the previous 4 weeks; and treatment with any erythropoiesis stimulating agents (ESA) within the previous 12 weeks.

### Randomization and dosing

We used dynamic balanced randomization to 1:1 randomize patients to either FC-low group (500 mg, approximately 120 mg elemental iron/day) or FC-high group (1000 mg, approximately 240 mg elemental iron/day) while ensuring balance of baseline Hb level and presence or absence of CKD across the two groups. FC was administered orally once a day in the FC-low group and twice a day in the FC-high group immediately after meals.

If sufficient iron replacement had been achieved after week 8, further treatment of FC was discontinued. Iron replacement was suggested to be sufficient if serum ferritin increased to ≥50.0 ng/mL in CKD patients and to ≥25.0 ng/mL in non-CKD patients.

The use of the following drugs was prohibited during the study: (1) oral or intravenous irons (2) ESAs, (3) protein anabolic hormones, testosterone enanthate, and mepitiostane, (4) drugs intended to improve absorption of oral irons, (5) drugs intended for the treatment of hyperphosphatemia.

### Laboratory assessments

Anemia-related parameters were assessed at baseline, weeks 4, and 8, and end of treatment (EOT, week 24 or after the day of discontinuation), including iron parameters [serum iron, serum ferritin, total iron-binding capacity (TIBC), TSAT, hepcidin-25, soluble transferrin receptor (sTfR)], and erythrocyte parameters [Hb, red blood cell count, hematocrit, mean corpuscular volume (MCV), mean corpuscular hemoglobin (MCH), mean corpuscular hemoglobin concentration (MCHC), red blood cell distribution width (RDW), reticulocyte count]. iFGF23 and cFGF23 levels were assessed at baseline, week 8, and EOT.

Serum phosphorus, serum calcium, PLT, quantitative C-reactive protein (CRP), prothrombin time-international normalized ratio (PT-INR), activated partial thromboplastin time (APTT), and fibrinogen were assessed at baseline, weeks 4, and 8, and EOT.

To standardize the measurement methods, all clinical laboratory tests were collectively performed at a central laboratory. Levels of serum iFGF23 (full-length), plasma cFGF23 (full-length plus C-terminal cleavage fragment), serum hepcidin-25 and serum sTfR were measured by FGF23 ELISA Kit (Kainos, Tokyo, Japan), Human FGF23 (C-Term) ELISA Kit (Immutopics, Inc., CA, USA), Quantikine® ELISA Human Hepcidin Immunoassay (R&D Systems Inc., MN, USA) and Access sTfR assay in Access immunoassay system (Beckman Coulter Inc., CA, USA), respectively.

### Statistical analysis

The target sample size was set at 35 patients per group (a total of 70) to evaluate the efficacy of FC as iron replacement therapy [22]. Patients who received study treatment and had at least one assessment for efficacy were included in the modified intention-to-treat population; the safety analysis population included participants with at least one safety assessment. Within these populations, we calculated changes from baseline to week 8 and EOT, and their 95% confidence intervals. For analyses of PLT, the upper normal range of PLT was defined as 35.2 × 10^4^/µL (the 97.5% upper reference limit in healthy adult in Japan) [25] and 45.0 × 10^4^/μL (the WHO definition of thrombocytosis) [26]. All statistical analyses were conducted using SAS version 9.4 (SAS Institute Inc., Cary, NC, USA), and all adverse events were categorized using standardized terminology by MedDRA version 21.0.

## Results

### Patient disposition and baseline characteristics

**Fig. 1:**
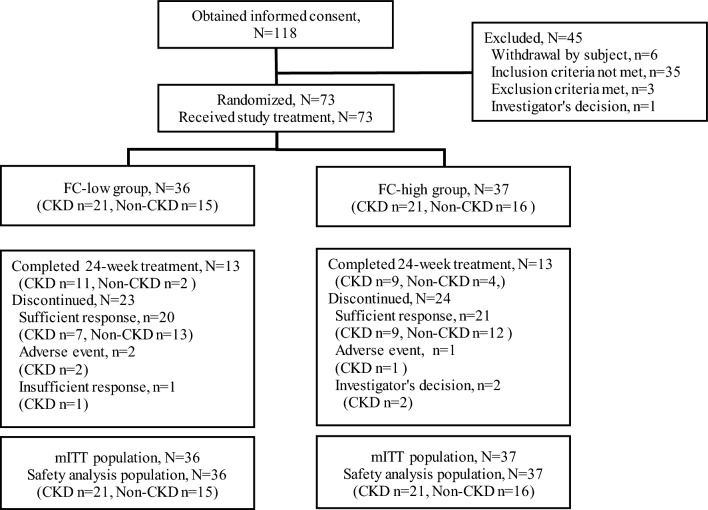
Disposition of patient. *mITT* Modified intention-to-treat population

Thirty-six patients were randomized to the FC-low group (CKD *n* = 21, non-CKD *n* = 15), and 37 patients were randomized to the FC-high group (CKD *n* = 21, non-CKD *n* = 16; Table 1 and Fig. 1). Across the two groups, 72 of 73 patients (98.6%) completed 8 weeks of treatment and 26 patients (35.6%) completed the full 24-week treatment; 47 patients (64.4%) discontinued in total, because they had achieved sufficient iron replacement; 41 patients (56.2%) or for other reasons (Fig. 1). Mean treatment duration ± standard deviation (SD) in the FC-low group was 127.0 ± 47.1 days in CKD patients and 108.1 ± 30.7 days in non-CKD patients; in the FC-high group was 119.6 ± 43.1 days in CKD patients and 107.6 ± 38.8 days in non-CKD patients. Adverse events leading to withdrawal from the FC-low group (*n* = 2) were constipation and diarrhoea, hepatic enzyme increased and malaise, and abdominal distension led to withdrawal from the FC-high group (*n* = 1). All patients (*n* = 73) were included in the modified intention-to-treat and safety populations (Fig. 1).

**Table 1:** Patient demographics (modified intention-to-treat population)

Characteristics	FC-low (*n* = 36)		FC-high (*n* = 37)	
	CKD (*n* = 21)	Non-CKD (*n* = 15)	CKD (*n* = 21)	Non-CKD (*n* = 16)
Age, years, mean ± SD	73.2 ± 14.0	45.2 ± 7.1	67.7 ± 13.2	46.7 ± 8.6
Sex, *n *(%)				
Male	7 (33.3)	0 (0.0)	4 (19.0)	0 (0.0)
Female	14 (66.7)	15 (100.0)	17 (81.0)	16 (100.0)
Menopausal status^a^, *n* (%)				
Pre-menopause	2 (14.3)	15 (100.0)	5 (29.4)	15 (93.8)
Post-menopause	12 (85.7)	0 (0.0)	12 (70.6)	1 (6.3)
Primary cause of IDA^b^, *n* (%)				
Uterine myoma	0 (0.0)	3 (20.0)	0 (0.0)	5 (31.3)
Adenomyosis uteri	0 (0.0)	4 (26.7)	0 (0.0)	1 (6.3)
Endometriosis	0 (0.0)	0 (0.0)	0 (0.0)	1 (6.3)
Other	1 (4.8)	4 (26.7)	1 (4.8)	2 (12.5)
Unknown	20 (95.2)	4 (26.7)	20 (95.2)	9 (56.3)
eGFR, mean ± SD (mL/min/1.73 m^2^)	41.2 ± 12.4	84.5 ± 14.3	41.8 ± 14.7	90.0 ± 14.7
Underlying cause of CKD^b^ (*n*, %)				
Diabetic nephropathy	9 (42.9)	–	6 (28.6)	–
Chronic glomerulonephritis	0 (0.0)	–	3 (14.3)	–
Nephrosclerosis	8 (38.1)	–	9 (42.9)	–
Unknown	5 (23.8)	–	3 (14.3)	–
Other	0 (0.0)	–	5 (23.8)	–

*FC-low group* ferric citrate hydrate at 500 mg (approximately 120 mg elemental iron)/day, *FC-high group* ferric citrate hydrate at 1000 mg (approximately 240 mg elemental iron)/day, *SD* standard deviation, *IDA* iron deficiency anemia, *CKD* chronic kidney disease, *eGFR* estimated glomerular filtration rate (creatinine-based)

^a^Only in women; ^b^Multiple choices allowed

### Iron, anemia, FGF23, and serum phosphorus

In both groups, regardless of CKD status, mean levels of serum iron, serum ferritin, TSAT, and hepcidin-25 increased, TIBC and sTfR decreased, and mean levels of Hb, red blood cell count, hematocrit, MCV, MCH and MCHC increased from baseline to week 8 and EOT (Table 2 and Supplementary file 1). Additionally, regardless of CKD status, mean levels of RDW and reticulocyte count tended to increase from baseline to up to 4 weeks in both groups (Supplementary file 2).

**Table 2:** Time course of iron-related parameters and hemoglobin (modified intention-to-treat population)

Parameter		BL Mean ± SD	Week 8 Mean ± SD	EOT Mean ± SD	Change: BL to week 8 Mean ± SD (95% CI)	Change: BL to EOT Mean ± SD (95% CI)
Serum iron (μg/dL)						
CKD	FC-low (*n* = 21)^a^	46.0 ± 24.5	70.0 ± 28.0	72.5 ± 25.2	23.1 ± 31.1, (8.5, 37.7)	26.5 ± 24.7, (15.3, 37.8)
	FC-high (*n* = 21)	45.2 ± 23.8	67.6 ± 24.6	74.8 ± 24.1	22.3 ± 25.9, (10.6, 34.1)	29.6 ± 27.3, (17.1, 42.0)
Non-CKD	FC-low (*n* = 15)	19.3 ± 14.9	63.9 ± 27.4	82.0 ± 22.6	44.6 ± 34.3, (25.6, 63.6)	62.7 ± 28.2, (47.1, 78.4)
	FC-high (*n* = 16)	15.7 ± 4.0	83.4 ± 69.7	93.1 ± 84.3	67.8 ± 71.0, (29.9, 105.6)	77.4 ± 85.8, (31.7, 123.1)
Serum ferritin (ng/mL)						
CKD	FC-low (*n* = 21)^a^	16.9 ± 9.1	35.8 ± 16.9	49.7 ± 20.6	18.8 ± 11.7, (13.3, 24.2)	32.9 ± 16.1, (25.5, 40.2)
	FC-high (*n* = 21)	15.9 ± 8.1	44.0 ± 33.5	69.2 ± 57.5	28.1 ± 32.2, (13.4, 42.7)	53.3 ± 54.7, (28.4, 78.1)
Non-CKD	FC-low (*n* = 15)	5.2 ± 2.6	22.7 ± 7.1	30.3 ± 8.5	17.5 ± 6.8, (13.8, 21.3)	25.2 ± 8.2, (20.6, 29.7)
	FC-high (*n* = 16)	4.9 ± 1.9	20.8 ± 7.1	26.7 ± 14.0	15.9 ± 7.2, (12.1, 19.8)	21.8 ± 14.3, (14.2, 29.5)
TIBC (μg/dL)						
CKD	FC-low (*n* = 21)^a^	382.3 ± 67.1	334.1 ± 53.5	318.2 ± 53.0	−46.0 ± 39.4, (−64.4, −27.5)	−64.1 ± 38.4, (−81.6, −46.6)
	FC-high (*n* = 21)	378.5 ± 58.5	328.1 ± 66.0	310.2 ± 66.1	−50.3 ± 37.8, (−67.5, −33.1)	−68.2 ± 38.6, (−85.8, −50.7)
Non-CKD	FC-low (*n* = 15)	448.2 ± 48.8	354.5 ± 31.7	344.5 ± 39.0	−93.7 ± 38.2, (−114.8, −72.5)	−103.7 ± 35.7, (−123.5, −83.9)
	FC-high (*n* = 16)	436.9 ± 51.9	356.6 ± 35.8	350.2 ± 43.6	−80.3 ± 47.5, (−105.6, −55.0)	−86.7 ± 50.4, (−113.5, −59.8)
TSAT (%)						
CKD	FC-low (*n* = 21)^a^	12.4 ± 6.7	20.8 ± 7.2	22.8 ± 7.3	8.1 ± 8.0, (4.4, 11.8)	10.3 ± 6.2, (7.5, 13.2)
	FC-high (*n* = 21)	12.5 ± 7.8	21.3 ± 8.1	25.0 ± 8.7	8.8 ± 7.4, (5.4, 12.1)	12.5 ± 9.0, (8.4, 16.6)
Non-CKD	FC-low (*n* = 15)	4.3 ± 3.5	18.1 ± 7.4	24.3 ± 7.8	13.8 ± 9.2, (8.7, 18.9)	19.9 ± 8.8, (15.1, 24.8)
	FC-high (*n* = 16)	3.6 ± 1.3	23.5 ± 18.8	26.6 ± 21.0	19.9 ± 19.0, (9.8, 30.1)	23.1 ± 21.3, (11.7, 34.4)
Hepcidin-25 (ng/mL)						
CKD	FC-low (*n* = 21)^a^	2.6 ± 3.6	10.9 ± 8.5	17.0 ± 9.8	8.3 ± 6.9, (5.1, 11.6)	14.5 ± 7.7, (11.0, 18.0)
	FC-high (*n* = 21)	2.9 ± 3.5	15.1 ± 10.4	23.5 ± 16.5	12.2 ± 10.4, (7.5, 17.0)	20.6 ± 14.6, (13.9, 27.2)
Non-CKD	FC-low (*n* = 15)	0.3 ± 0.6	7.9 ± 7.9	7.5 ± 6.0	7.6 ± 7.8, (3.3, 11.9)	7.2 ± 5.8, (3.9, 10.4)
	FC-high (*n* = 16)	0.2 ± 0.1	5.5 ± 4.9	9.2 ± 8.1	5.3 ± 4.9, (2.7, 7.9)	9.0 ± 8.0, (4.7, 13.3)
sTfR (nmol/L)						
CKD	FC-low (*n* = 21)^a^	24.3 ± 9.6	19.2 ± 4.4	17.2 ± 5.2	−5.1 ± 6.5, (−8.1, −2.0)	−7.1 ± 6.2, (−9.9, −4.3)
	FC-high (*n* = 21)	28.7 ± 18.3	18.8 ± 6.0	17.2 ± 4.8	−10.0 ± 13.5, (−16.1, −3.8)	−11.5 ± 15.5, (−18.6, −4.5)
Non-CKD	FC-low (*n* = 15)	44.4 ± 14.0	18.9 ± 5.0	16.7 ± 4.8	−25.5 ± 10.8, (−31.5, −19.5)	−27.7 ± 12.1, (−34.3, −21.0)
	FC-high (*n* = 16)	44.5 ± 14.9	22.0 ± 6.4	21.5 ± 8.7	−22.5 ± 14.8, (−30.4, −14.6)	−23.0 ± 17.6, (−32.4, −13.6)
Hemoglobin (g/dL)						
CKD	FC-low (*n* = 21)^a^	10.2 ± 0.8	11.4 ± 0.7	11.8 ± 1.0	1.3 ± 1.1, (0.8, 1.8)	1.6 ± 1.3, (1.0, 2.2)
	FC-high (*n* = 21)	10.2 ± 0.6	11.9 ± 1.3	12.0 ± 1.4	1.7 ± 1.6, (0.9, 2.4)	1.8 ± 1.8, (1.0, 2.6)
Non-CKD	FC-low (*n* = 15)	9.4 ± 0.6	12.4 ± 0.8	12.9 ± 0.9	3.0 ± 0.9, (2.5, 3.5)	3.5 ± 1.0, (2.9, 4.0)
	FC-high (*n* = 16)	9.4 ± 0.6	12.4 ± 1.2	12.8 ± 1.7	3.0 ± 1.3, (2.3, 3.7)	3.4 ± 1.8, (2.4, 4.3)

*BL* baseline, *CI* confidence interval, *CKD* chronic kidney disease, *FC-low group* ferric citrate hydrate at 500 mg (approximately 120 mg elemental iron)/day, *FC-high group* ferric citrate hydrate at 1000 mg (approximately 240 mg elemental iron)/day, *EOT* end of treatment, *TIBC* total iron-binding capacity, *TSAT* transferrin saturation, *sTfR* soluble transferrin receptor, *SD* standard deviation

^a^Week 8, *n* = 20

At baseline, median iFGF23 (pg/mL) levels tended to be higher in CKD versus non-CKD patients, whereas median cFGF23 (RU/mL) levels tended to be lower in CKD than non-CKD patients (Table 3).

**Table 3:** Time course of fibroblast growth factor 23 (modified intention-to-treat population)

Parameters		BL Median (Q1, Q3)	Week 8 Median (Q1, Q3)	EOT Median (Q1, Q3)	Change from BL to week 8 Median (Q1, Q3)	95% CI^b^ (BL to week 8)	Change from BL to EOT Median (Q1, Q3)	95% CI^b^ (BL to EOT)
Intact FGF23 (pg/mL)								
CKD	FC-low (*n* = 21)^a^	64.00 (48.10, 82.20)	63.00 (53.70, 77.65)	62.10 (52.00, 74.60)	1.00 (−9.35, 13.90)	−9.30, 13.40	2.60 (−10.10, 14.40)	−10.10, 14.40
	FC-high (*n* = 21)	58.20 (45.10, 72.50)	54.50 (49.60, 68.90)	56.30 (48.10, 72.10)	0.90 (−11.10, 7.30)	−11.10, 7.30	1.30 (−7.10, 5.20)	−7.10, 5.20
Non-CKD	FC-low (*n* = 15)	40.80 (37.50, 47.20)	41.70 (35.10, 49.10)	37.40 (30.90, 51.30)	−2.60 (−6.50, 5.40)	−6.50, 5.40	−3.90 (−11.70, 6.00)	−11.70, 6.00
	FC-high (*n* = 16)	35.90 (30.10, 48.75)	37.85 (28.00, 43.35)	39.05 (29.90, 45.35)	−1.90 (−7.10, 2.65)	−5.80, 3.20	−3.40 (−5.20, 5.15)	−5.10, 5.30
C-terminal FGF23 (RU/mL)								
CKD	FC-low (n = 21)^a^	159.00 (135.00, 390.00)	108.50 (85.25, 132.00)	104.00 (90.40, 118.00)	−58.00 (−227.50, −12.25)	−181.00, −12.50	−71.90 (−181.00, −11.00)	−181.00, −11.00
	FC-high (*n* = 21)	188.0 (136.00, 361.00)	110.00 (91.50, 135.00)	114.00 (89.30, 153.00)	−66.00 (−265.70, −27.00)	−265.70, −27.00	−74.00 (−265.70, −18.90)	−265.7, −18.9
Non-CKD	FC-low (*n *= 15)	1010.00 (260.00, 1240.00)	102.00 (83.60, 122.00)	96.10 (73.00, 155.00)	−725.00 (−1124.00, −168.50)	−1124.00, −168.50	−745.00 (−1124.00, −183.00)	−1124.00, −183.00
	FC-high (*n* = 16)	775.00 (394.00, 1285.00)	104.50 (65.50, 138.00)	104.50 (70.75, 144.00)	−649.50 (−1127.00, −326.65)	−988.00, −299.40	−666.50 (−1191.50, −319.40)	−1117.00, −298.40

*BL* baseline, *CI* confidence interval, *CKD* chronic kidney disease, *FGF23* fibroblast growth factor 23 *FC-low group* ferric citrate hydrate at 500 mg (approximately 120 mg elemental iron)/day, *FC-high group* ferric citrate hydrate at 1000 mg (approximately 240 mg elemental iron)/day, *EOT* end of treatment

^a^Week 8, *n* = 20; ^b^95% CI for median

**Fig. 2:**
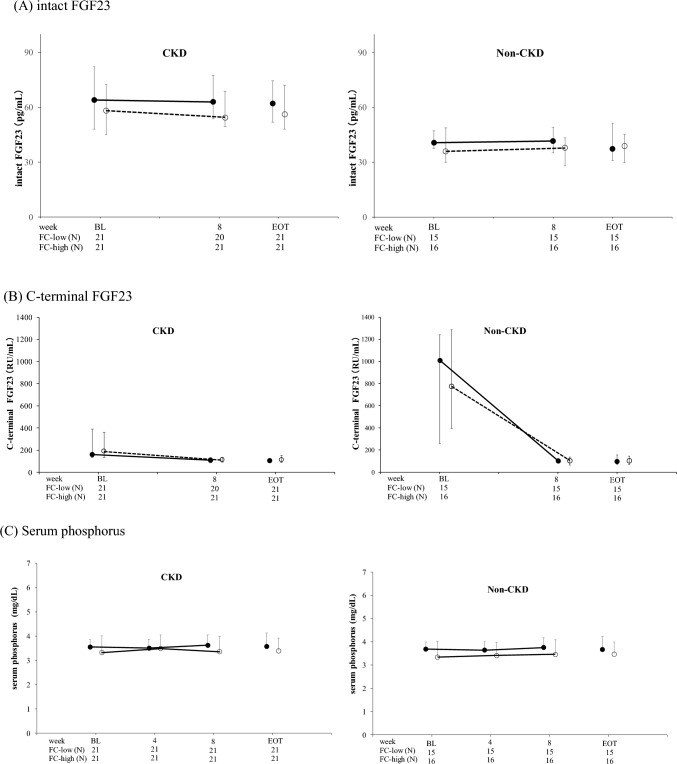
Time course of intact FGF23, C-terminal FGF23, and serum phosphorus levels. **A** Time course of intact FGF23 in CKD and non-CKD patients. **B** Time course of C-terminal FGF23 in CKD and non-CKD patients. **C** Time course of serum phosphorus in CKD and non-CKD patients. FC-low group (black circles); ferric citrate hydrate at 500 mg (approximately 120 mg elemental iron)/day, FC-high group (white circles); ferric citrate hydrate at 1000 mg (approximately 240 mg elemental iron)/day, *BL* baseline, *EOT* end of treatment, Data are presented as median (Q1, Q3) in FGF23 and mean ± standard deviation in serum phosphorus

After administration of FC, median iFGF23 and mean serum phosphorus levels did not change regardless of CKD status (Tables 3 and 4; Fig. 2(A), (C)), whereas in both groups, median cFGF23 levels decreased at week 8 regardless of CKD status (Tables 3; Fig. 2(B)).

**Table 4:** Time courses of serum phosphorus, platelet count, serum calcium and CRP (safety analysis population)

Parameters		BL Mean ± SD	Week 8 Mean ± SD	EOT Mean ± SD	Change from BL to week 8 Mean ± SD	95% CI (BL to week 8)	Change from BL to EOT Mean ± SD	95% CI (BL to EOT)
Serum phosphorus (mg/dL)								
CKD	FC-low (*n* = 21)^a^	3.56 ± 0.72	3.63 ± 0.49	3.57 ± 0.43	0.11 ± 0.60	−0.18, 0.39	0.01 ± 0.58	−0.25, 0.27
	FC-high (*n* = 21)	3.32 ± 0.48	3.36 ± 0.42	3.40 ± 0.51	0.03 ± 0.44	−0.17, 0.23	0.08 ± 0.48	−0.14, 0.30
Non-CKD	FC-low (*n* = 15)	3.69 ± 0.30	3.75 ± 0.42	3.67 ± 0.56	0.06 ± 0.50	−0.21, 0.33	−0.02 ± 0.63	−0.37, 0.33
	FC-high (*n* = 16)	3.34 ± 0.69	3.46 ± 0.63	3.47 ± 0.52	0.12 ± 0.57	−0.19, 0.42	0.13 ± 0.60	−0.19, 0.45
Platelet count (10^4^/μL)								
CKD	FC-low (*n* = 21)^a^	24.27 ± 7.17	21.89 ± 5.25	21.58 ± 6.22	−1.78 ± 4.05	−3.67, 0.11	−2.69 ± 4.10	−4.56, −0.82
	FC-high (*n* = 21)^b^	26.56 ± 8.58	25.09 ± 6.97	23.69 ± 6.62	−1.56 ± 7.28	−5.07, 1.95	−2.87 ± 4.58	−5.01, −0.72
Non-CKD	FC-low (*n* = 15)	33.25 ± 9.02	24.35 ± 5.47	24.91 ± 6.95	−8.91 ± 5.36	−11.88, −5.94	−8.34 ± 5.32	−11.28, −5.40
	FC-high (*n* = 16)	36.99 ± 8.38	29.58 ± 4.41	28.63 ± 4.64	− 7.41 ± 6.25	−10.74, −4.08	−8.36 ± 7.34	−12.27, −4.45
Serum calcium (mg/dL)								
CKD	FC-low (*n* = 21)^a^	9.22 ± 0.70	9.34 ± 0.48	9.25 ± 0.56	0.16 ± 0.43	−0.05, 0.36	0.03 ± 0.41	−0.15, 0.22
	FC-high (*n* = 21)	9.00 ± 0.31	9.23 ± 0.45	9.10 ± 0.34	0.23 ± 0.32	0.09, 0.38	0.11 ± 0.25	−0.01, 0.23
Non-CKD	FC-low (*n* = 15)	9.04 ± 0.14	9.18 ± 0.34	9.10 ± 0.43	0.14 ± 0.31	−0.03, 0.31	0.06 ± 0.43	−0.18, 0.30
	FC-high (*n *= 16)	9.08 ± 0.28	9.27 ± 0.25	9.22 ± 0.24	0.19 ± 0.23	0.07, 0.31	0.14 ± 0.32	−0.04, 0.31
CRP (mg/dL)								
CKD	FC-low (*n* = 21)^a^	0.14 ± 0.23	0.14 ± 0.14	0.15 ± 0.30	0.01 ± 0.20	−0.09, 0.10	0.01 ± 0.33	−0.14, 0.16
	FC-high (*n* = 21)	0.23 ± 0.63	0.34 ± 0.94	0.39 ± 0.80	0.11 ± 1.15	−0.42, 0.63	0.16 ± 1.03	−0.31, 0.63
Non-CKD	FC-low (*n* = 15)	0.14 ± 0.44	0.15 ± 0.40	0.14 ± 0.41	0.01 ± 0.08	−0.03, 0.05	0.003 ± 0.03	−0.02, 0.02
	FC-high (*n* = 16)	0.08 ± 0.09	0.06 ± 0.08	0.07 ± 0.07	−0.02 ± 0.08	−0.06, 0.03	−0.01 ± 0.07	−0.05, 0.03

*BL* baseline, *CI* confidence interval, *CKD* chronic kidney disease, *CRP* C-reactive protein, *FC-low group* ferric citrate hydrate at 500 mg (approximately 120 mg elemental iron)/day, *FC-high group* ferric citrate hydrate at 1000 mg (approximately 240 mg elemental iron)/day, *EOT* end of treatment, *SD* standard deviation

^a^Week 8, *n* = 20; ^b^Week 8, *n* = 19

### PLT, serum calcium, CRP, and coagulation

**Fig. 3:**
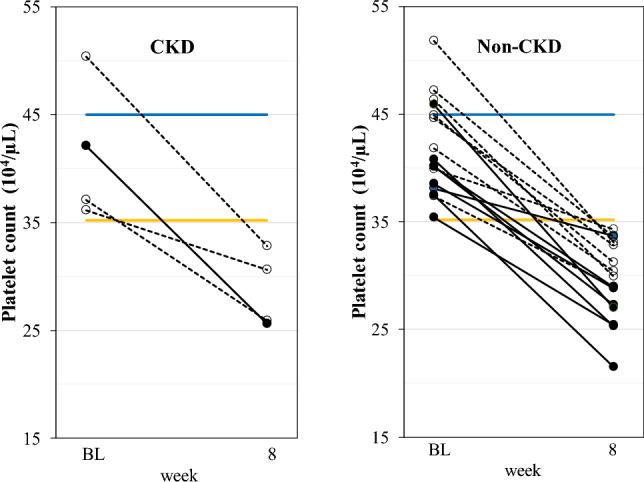
Changes in platelet count in patients with high platelet count at baseline. FC-low group (black circles); ferric citrate hydrate at 500 mg (approximately 120 mg elemental iron)/day, FC-high group (white circles); ferric citrate hydrate at 1000 mg (approximately 240 mg elemental iron)/day), *BL* baseline, Yellow line is 35.2 × 10^4^/µL and blue line is 45.0 × 10^4^/µL

At baseline, high PLT (>35.2 × 10^4^/µL) was observed in one (5.0%) CKD patient and eight (53.3%) non-CKD patients in the FC-low group, and three (15.8%) CKD patients and eight (50.0%) non-CKD patients in the FC-high group. Similarly, at baseline high PLT (>45.0 × 10^4^/µL) was observed in one (6.7%) non-CKD patient in the FC-low group, and one (5.3%) CKD patient and three (18.8%) non-CKD patients in the FC-high group. After administration of FC, mean PLT decreased from baseline to week 8 and EOT in non-CKD patients, as well as from baseline to EOT in CKD patients (Table 4). However, no clinically meaningful changes were observed in patients without high PLT at baseline both in CKD and non-CKD patients (Supplementary file 3). Regardless of CKD status, FC decreased PLT to below 35.2 × 10^4^/µL by week 8 in all patients with high PLT at baseline (Fig. 3).

In both groups, FC administration did not change mean levels of serum calcium and CRP, PT-INR, APTT or fibrinogen (Table 4, Supplementary file 4).

## Discussion

Iron deficiency increases cFGF23 levels and PLT, and each of which is associated with increased cardiovascular risk [3–6, 13, 14, 27, 28]. We had hypothesized that iron replacement with FC would decrease elevated cFGF23 levels and normalize high PLT in NDD-CKD and non-CKD patients with IDA.

Mean levels of RDW and reticulocyte count tended to increase up to week 4 after FC administration. The increase in reticulocyte count after oral iron administration is an indirect marker of functional iron utilization for erythropoiesis [29]. The increase in RDW level reflects increased reticulocyte count, because reticulocytes are larger than mature red blood cells. As increases in mean levels of Hb were also observed, iron replacement with FC was successfully utilized for erythropoiesis regardless of CKD status.

In CKD, iFGF23 and cFGF23 levels increase progressively from the early stages of CKD to maintain normal serum phosphorus levels [30]. As expected, in the present study, baseline median iFGF23 levels were higher in CKD versus non-CKD patients. Interestingly, median levels of cFGF23 were lower at baseline in CKD patients versus non-CKD patients. It is possible that there are two reasons for this unexpected finding. Firstly, IDA was more severe at baseline in non-CKD patients compared with CKD patients, as suggested by their lower TSAT, serum ferritin, and Hb levels. More severe IDA would result in more stimulation of FGF23 production and cleavage. Secondarily, as previous studies showed that FGF23 degradation was suppressed in CKD compared with non-CKD in mice and humans to retain serum phosphorus levels [31, 32]. FGF23 cleavage was likely also more suppressed in CKD patients than in non-CKD patients in the present study. From the above reasons, IDA-induced C-terminal cleavage fragments might be smaller in CKD patients than in non-CKD patients. It would result in lower levels of cFGF23 in CKD patients than in non-CKD patients at baseline.

Unlike cFGF23, FC did not affect iFGF23 levels in either CKD or non-CKD patients, likely because FC decreased elevated production and cleavage of FGF23 as well as elevated C-terminal cleavage fragments by correcting iron deficiency. This resulted in a decrease in cFGF23 levels without altering the need for iFGF23 levels. With no change in iFGF23 levels, there were also no changes in serum phosphorus in either group regardless of CKD status.

Intravenous FCM decreases cFGF23 levels but simultaneously increases iFGF23 levels, which can cause hypophosphatemia [33]. In contrast, an increase in iFGF23 levels and subsequent decreases in serum phosphorus levels were not observed in oral FC in the present study. In a previous study that compared the effects of oral sodium ferrous citrate versus intravenous saccharated ferric oxide on FGF23 levels in hemodialysis patients with iron deficiency, serum phosphorus levels did not change during the study, whereas iFGF23 levels increased in the intravenous iron group but did not change in the oral iron group; cFGF23 levels decreased in both groups [34]. Based on these data and the current results, iron replacement with oral irons, including FC, is not likely to increase iFGF23 levels and thus should not cause hypophosphatemia, unlike intravenous irons.

In another previous study in which FC or oral sodium ferrous citrate was administered to NDD-CKD patients with iron deficiency [35], iFGF23 and cFGF23 levels did not change. Unlike the previous study, the decrease in cFGF23 in the present study might be attributable to current patients having more severe iron deficiency at baseline. Additionally, plasma is the optimal collection method for cFGF23 assays because serum can underestimate the measurement [36]. Therefore, the plasma levels used in the present study could have measured cFGF23 more sensitively than in the prior study that used serum. The other previous study of oral sodium ferrous citrate in hemodialysis patients with iron deficiency reported a decrease in iFGF23, cFGF23, dose of ESA, and CRP levels without changes in serum phosphorus [37]. It is suggested that inflammation and increased erythropoietin increase production and cleavage of FGF23 [38]. The use of ESA was prohibited, and CRP levels did not change, and therefore iFGF23 level may not have changed in the present study.

An observational study in the general population reported that higher cFGF23 levels were associated with higher risk of mortality [9]. In another observational study in patients with mild to moderate NDD-CKD, the prevalence and incidence of anemia were significantly higher in patients with higher baseline cFGF23 levels [39] and cFGF23 levels mediated the increased risks of mortality and heart failure attributable to iron deficiency [40]. Higher cFGF23 levels are associated with increased risks of mortality and heart failure, which may be driven by direct, toxic effects of FGF23 on the heart [41]. In a mouse model of CKD, ferric citrate (Auryxia®) improved cardiac function and prolonged life span [42]. In conjunction with the result of the present study, iron replacement with FC might be expected to reduce the risks of cardiovascular events and mortality by decreasing elevated cFGF23 levels in patients with IDA.

Although the detailed mechanism by which PLT increases in some patients with IDA is incompletely understood, iron deficiency has been shown to affect the differentiation of megakaryocyte-erythrocyte progenitors in the bone marrow and to increase lineage-commitment to the megakaryocyte lineage in humans and mice [43]. In the present study, some patients with elevated PLT at baseline were observed as well, and the percentage of patients with elevated PLT at baseline was lesser in CKD patients than in non-CKD patients. Erythropoietin acts on hematopoietic stem cells in the bone marrow to stimulate blood cell production, and erythropoietin secretion decreases in CKD patients, which could be one of the reasons why the degree of PLT elevation at baseline was lesser in CKD patients than in non-CKD patients. By contrast, in a community-based study, relative risk of venous thromboembolism was higher in NDD-CKD participants than in non-CKD participants [44]. Compared to the previous study of oral ferrous succinate in non-CKD patients with IDA [17], FC normalized PLT in patients with higher PLT (>35.2 × 10^4^/µL or >45.0 × 10^4^/µL) at baseline not only in non-CKD patients but also in NDD-CKD patients with IDA. Even though it is speculative at present, our results suggest that iron replacement with FC in patients with IDA normalized the imbalanced differentiation of hematopoietic stem cells caused by iron deficiency. Given that higher PLT in patients with IDA increases risk of thrombosis [14, 28], administration of FC in IDA patients with higher PLT might be expected to reduce the risk by reducing PLT into the normal range.

Limitations of this study include the small number of patients, because the goal of the primary study was to investigate the iron replacement effect of FC rather than the effects on FGF23 and PLT. Additional larger studies are warranted to establish the effect of FC on FGF23 and PLT. Additionally, we only measured FGF23 at a limited number of time points. As intravenous iron altered FGF23 within 24 h after administration [33], data on more early time points after administration of FC would be of interest.

In conclusion, in both NDD-CKD and non-CKD patients with IDA, administration of oral FC increased serum ferritin and TSAT, decreased cFGF23 without affecting levels of iFGF23 or serum phosphorus, and normalized PLT in patients with high PLT at baseline. Future studies should investigate whether these effects of FC might translate into decreased cardiovascular risk in patients with IDA.

### Supplementary Information

Below is the link to the electronic supplementary material.

Supplementary file1 (DOCX 34 kb)

Supplementary file2 (DOCX 116 kb)

Supplementary file3 (DOCX 77 kb)

Supplementary file4 (DOCX 30 kb)

## Data Availability

The data generated and/or analyzed in the present study are available from the corresponding author on reasonable request.
