# Supplementary material for: Effect of ferric citrate hydrate on fibroblast growth factor 23 and platelets in non-dialysis-dependent chronic kidney disease and non-chronic kidney disease patients with iron deficiency anemia
Source: Clin Exp Nephrol. 2024 Feb 25;28(7):636–46. doi: 10.1007/s10157-023-02455-6 (PMC11189996; doi:10.1007/s10157-023-02455-6)
Supplement: Supplementary file 1 — Supplementary file1 (DOCX 34 kb) [file 10157_2023_2455_MOESM1_ESM.docx]

**Supplementary file 1**
Time course of erythrocyte-related parameters ( modified intention-to-treat population)

| **Parameters** |  | **Baseline**  **Mean ± SD** | **Week 8**  **Mean ± SD** | **EOT**  **Mean ± SD** | **Change from baseline to week 8**  **Mean ± SD** | **95% CI**  **(baseline to week 8)** | **Change from baseline to EOT**  **Mean ± SD** | **95% CI**  **(baseline to EOT)** |
| --- | --- | --- | --- | --- | --- | --- | --- | --- |
| Red blood cell count (10^4^/μL) | |  |  |  |  |  |  |  |
| CKD | FC-low (n=21) ^a^ | 357.4 ± 30.6 | 381.5 ± 35.1 | 383.0 ± 38.4 | 25.3 ± 26.9 | 12.6, 37.9 | 25.6 ± 27.9 | 12.9, 38.3 |
|  | FC-high (n=21) | 382.6 ± 48.5 | 416.3 ± 67.6 | 405.7 ± 60.9 | 33.7 ± 31.6 | 19.3, 48.1 | 23.1 ± 26.7 | 11.0, 35.3 |
| Non-CKD | FC-low (n=15) | 416.3 ± 41.4 | 458.8 ± 37.0 | 443.8 ± 45.5 | 42.5 ± 28.6 | 26.6, 58.3 | 27.5 ± 56.3 | −3.7, 58.7 |
|  | FC-high (n=16) | 411.6 ± 39.3 | 455.2 ± 38.5 | 448.6 ± 41.9 | 43.6 ± 25.1 | 30.2, 57.0 | 37.1 ± 28.7 | 21.8, 52.4 |
| Hematocrit (%) |  |  |  |  |  |  |  |  |
| CKD | FC-low (n=21) ^a^ | 30.9 ± 2.3 | 34.4 ± 2.3 | 35.1 ± 3.2 | 3.5 ± 3.3 | 2.0, 5.1 | 4.2 ± 3.7 | 2.5, 5.9 |
|  | FC-high (n=21) | 31.2 ± 1.7 | 35.9 ± 4.1 | 35.9 ± 4.3 | 4.7 ± 4.5 | 2.7, 6.7 | 4.7 ± 4.7 | 2.6, 6.8 |
| Non-CKD | FC-low (n=15) | 29.8 ± 1.6 | 37.9 ± 2.3 | 38.6 ± 3.2 | 8.0 ± 2.4 | 6.7, 9.4 | 8.7 ± 3.5 | 6.8, 10.7 |
|  | FC-high (n=16) | 29.8 ± 1.8 | 37.8 ± 3.6 | 38.7 ± 4.7 | 8.0 ± 3.5 | 6.1, 9.9 | 8.8 ± 4.7 | 6.3, 11.3 |
| MCV (fL) |  |  |  |  |  |  |  |  |
| CKD | FC-low (n=21) ^a^ | 86.8 ± 5.5 | 90.4 ± 4.3 | 91.9 ± 4.2 | 1.6 ± 1.2 | 1.0, 2.1 | 2.3 ± 1.9 | 1.4, 3.2 |
|  | FC-high (n=21) | 82.6 ± 8.6 | 86.9 ± 4.7 | 88.9 ± 4.2 | 1.7 ± 2.0 | 0.8, 2.6 | 2.6 ± 2.7 | 1.4, 3.9 |
| Non-CKD | FC-low (n=15) | 72.3 ± 6.7 | 82.8 ± 5.6 | 87.3 ± 6.0 | 4.4 ± 1.9 | 3.4, 5.4 | 6.4 ± 3.0 | 4.8, 8.0 |
|  | FC-high (n=16) | 73.0 ± 5.8 | 82.9 ± 3.8 | 86.0 ± 5.6 | 4.2 ± 2.7 | 2.8, 5.6 | 5.4 ± 3.3 | 3.7, 7.2 |
| MCH (pg) |  |  |  |  |  |  |  |  |
| CKD | FC-low (n=21) ^a^ | 28.5 ± 2.2 | 30.1 ± 1.7 | 30.8 ± 1.7 | 3.5 ± 3.3 | 2.0, 5.0 | 5.0 ± 4.8 | 2.9, 7.2 |
|  | FC-high (n=21) | 27.1 ± 3.4 | 28.8 ± 1.8 | 29.7 ± 1.5 | 4.3 ± 5.1 | 2.0, 6.6 | 6.3 ± 6.4 | 3.4, 9.3 |
| Non-CKD | FC-low (n=15) | 22.8 ± 2.6 | 27.2 ± 1.9 | 29.2 ± 2.3 | 10.5 ± 4.5 | 8.1, 13.0 | 15.0 ± 6.7 | 11.3, 18.7 |
|  | FC-high (n=16) | 23.0 ± 2.3 | 27.2 ± 1.4 | 28.4 ± 2.1 | 9.9 ± 6.8 | 6.3, 13.6 | 13.0 ± 8.1 | 8.7, 17.3 |
| MCHC (%) |  |  |  |  |  |  |  |  |
| CKD | FC-low (n=21) ^a^ | 32.8 ± 0.6 | 33.3 ± 0.6 | 33.5 ± 0.7 | 0.4 ± 0.4 | 0.2, 0.6 | 0.7 ± 0.7 | 0.4, 1.0 |
|  | FC-high (n=21) | 32.7 ± 1.1 | 33.1 ± 0.5 | 33.4 ± 0.5 | 0.4 ± 0.9 | −0.02, 0.8 | 0.7 ± 1.1 | 0.2, 1.2 |
| Non-CKD | FC-low (n=15) | 31.5 ± 0.8 | 32.8 ± 0.5 | 33.4 ± 0.9 | 1.3 ± 0.8 | 0.9, 1.8 | 1.9 ± 1.3 | 1.2, 2.6 |
|  | FC-high (n=16) | 31.5 ± 0.7 | 32.7 ± 0.6 | 33.0 ± 0.6 | 1.2 ± 0.8 | 0.7, 1.6 | 1.5 ± 1.0 | 1.0, 2.0 |
| RDW (%) |  |  |  |  |  |  |  |  |
| CKD | FC-low (n=21) ^a^ | 15.2 ± 1.8 | 16.6 ± 2.8 | 14.8 ± 2.2 | 1.4 ± 1.6 | 0.7, 2.1 | −0.4 ± 1.5 | −1.1, 0.3 |
|  | FC-high (n=21) | 15.6 ± 2.1 | 17.7 ± 4.6 | 15.6 ± 3.0 | 2.2 ± 2.8 | 0.9, 3.4 | 0.02 ± 2.1 | −0.9, 1.0 |
| Non-CKD | FC-low (n=15) | 18.3 ± 2.9 | 23.5 ± 2.1 | 16.0 ± 3.2 | 5.2 ± 3.2 | 3.5, 7.0 | −2.3 ± 4.3 | −4.7, 0.1 |
|  | FC-high (n=16) | 18.0 ± 2.1 | 24.2 ± 4.3 | 18.4 ± 5.7 | 6.3 ± 3.1 | 4.6, 7.9 | 0.5 ± 5.5 | −2.4, 3.4 |
| Reticulocyte count (10^4^/μL) | |  |  |  |  |  |  |  |
| CKD | FC-low (n=21) ^a^ | 4.2 ± 1.3 | 5.1 ± 1.6 | 4.7 ± 1.9 | 0.9 ± 1.7 | 0.2, 1.8 | 0.6 ± 1.6 | −0.2, 1.3 |
|  | FC-high (n=21) | 5.3± 1.7 | 6.0 ± 2.0 | 6.0 ± 2.3 | 0.6 ± 1.8 | −0.2, 1.5 | 0.7 ± 2.2 | −0.3, 1.7 |
| Non-CKD | FC-low (n=15) | 6.1 ± 2.8 | 5.8 ± 3.2 | 6.5 ± 3.1 | −0.3 ± 2.7 | −1.8, 1.2 | 0.4 ± 2.0 | −0.7, 1.5 |
|  | FC-high (n=16) | 5.3 ± 2.3 | 6.3 ± 3.1 | 6.2 ± 2.7 | 0.9 ± 2.6 | −0.5, 2.3 | 0.9 ± 2.3 | −0.4, 2.2 |
| ^a^Week 8, n=20 | | | | | | | | |
| CI, confidence interval; CKD, chronic kidney disease; SD, standard deviation; FC-low group, ferric citrate hydrate at 500 mg (approximately 120 mg elemental iron)/day; FC-high group, ferric citrate hydrate at 1000 mg (approximately 240 mg elemental iron)/day; EOT, end of treatment; MCV, mean corpuscular volume; MCH, mean corpuscular haemoglobin; MCHC, mean corpuscular haemoglobin concentration; RDW, red blood cell distribution width | | | | | | | | |
