# Supplementary material for: Effect of ferric citrate hydrate on fibroblast growth factor 23 and platelets in non-dialysis-dependent chronic kidney disease and non-chronic kidney disease patients with iron deficiency anemia
Source: Clin Exp Nephrol. 2024 Feb 25;28(7):636–46. doi: 10.1007/s10157-023-02455-6 (PMC11189996; doi:10.1007/s10157-023-02455-6)
Supplement: Supplementary file 2 — Supplementary file2 (DOCX 116 kb) [file 10157_2023_2455_MOESM2_ESM.docx]

**Supplementary file 2**
Time course of red blood cell distribution width and reticulocyte count

1. Time course of red blood cell distribution width in CKD patients and non-CKD patients.
   FC-low group (black circles), FC-high group (white circles), from baseline to week 8, and EOT.

Data are presented as mean ± SD.


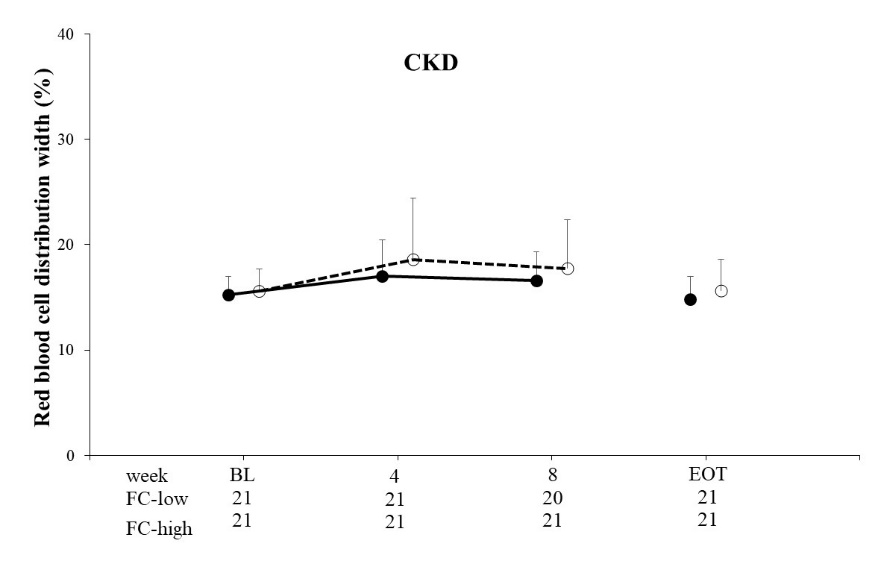


1. Time course of reticulocyte count in CKD patients and non-CKD patients.
   FC-low group (black circles), FC-high group (white circles), from baseline to weeks 4, and 8, and EOT.

Data are presented as mean ± SD.
