# Supplementary material for: Effect of ferric citrate hydrate on fibroblast growth factor 23 and platelets in non-dialysis-dependent chronic kidney disease and non-chronic kidney disease patients with iron deficiency anemia
Source: Clin Exp Nephrol. 2024 Feb 25;28(7):636–46. doi: 10.1007/s10157-023-02455-6 (PMC11189996; doi:10.1007/s10157-023-02455-6)
Supplement: Supplementary file 3 — Supplementary file3 (DOCX 77 kb) [file 10157_2023_2455_MOESM3_ESM.docx]

**Supplementary file 3**
Changes in platelet count in patients without high platelet count (>35.2 × 10^4^/µL) at baseline

FC-low group (black circles), FC-high group (white circles), from baseline to week 8.

Yellow lines are 35.2 × 10^4^/µL and 16.0× 10^4^/µL which are 97.5% upper reference limit and 2.5% lower reference limit in healthy adult in Japan, respectively [25]

Blue line are 45.0 × 10^4^/µL and 15.0 × 10^4^/µL which are WHO definition of thrombocytosis and thrombocytopenia, respectively [26, 45]

FC-low group, ferric citrate hydrate at 500 mg (approximately 120 mg elemental iron)/day;
FC-high group, ferric citrate hydrate at 1000 mg (approximately 240 mg elemental iron)/day.
