# Supplementary material for: Effect of ferric citrate hydrate on fibroblast growth factor 23 and platelets in non-dialysis-dependent chronic kidney disease and non-chronic kidney disease patients with iron deficiency anemia
Source: Clin Exp Nephrol. 2024 Feb 25;28(7):636–46. doi: 10.1007/s10157-023-02455-6 (PMC11189996; doi:10.1007/s10157-023-02455-6)
Supplement: Supplementary file 4 — Supplementary file4 (DOCX 30 kb) [file 10157_2023_2455_MOESM4_ESM.docx]

**Supplementary file 4** Time course in coagulation-related parameters (safety analysis population)

| Parameters |  | **Baseline**  **Mean ± SD** | **Week 8**  **Mean ± SD** | **EOT**  **Mean ± SD** | **Change from baseline to week 8**  **Mean ± SD** | **95% CI**  **(baseline to week 8)** | **Change from baseline to EOT**  **Mean ± SD** | **95% CI**  **(baseline to EOT)** |
| --- | --- | --- | --- | --- | --- | --- | --- | --- |
| PT-INR |  |  |  |  |  |  |  |  |
| CKD | FC-low (n=21) ^a^ | 1.07 ± 0.25 | 1.03 ±0.19 | 1.03 ±0.16 | −0.04 ± 0.11 | −0.09, 0.02 | −0.04 ±0.13 | −0.11, 0.02 |
|  | FC-high (n=21) | 1.08 ±0.35 | 1.14 ±0.60 | 1.09 ± 0.43 | 0.07 ± 0.27 | −0.06, 0.19 | 0.01 ± 0.14 | −0.05, 0.08 |
| Non-CKD | FC-low (n=15) | 0.99 ± 0.04 | 0.96 ± 0.05 | 0.96 ± 0.05 | −0.03 ± 0.07 | −0.07, 0.01 | −0.03 ± 0.06 | −0.06, 0.003 |
|  | FC-high (n=16) | 0.97 ± 0.04 | 0.97 ± 0.06 | 0.97 ± 0.04 | 0.001 ± 0.06 | −0.03, 0.03 | 0.003 ± 0.04 | −0.02, 0.02 |
| APTT (sec) |  |  |  |  |  |  |  |  |
| CKD | FC-low (n=21) ^a^ | 33.33 ± 4.10 | 33.39 ± 3.42 | 33.19 ± 3.73 | 0.27 ± 2.76 | −1.02, 1.55 | −0.15 ± 2.91 | −1.47, 1.18 |
|  | FC-high (n=21) | 33.02 ± 2.69 | 33.65 ± 3.54 | 33.23 ± 3.94 | 0.63 ± 2.60 | −0.55, 1.81 | 0.21 ± 2.49 | −0.93, 1.35 |
| Non-CKD | FC-low (n=15) | 31.01 ± 2.39 | 32.27 ± 3.22 | 32.17 ± 2.80 | 1.26 ± 2.44 | −0.09, 2.61 | 1.16 ± 2.32 | −0.12, 2.44 |
|  | FC-high (n=16) | 30.72 ± 3.28 | 32.73 ± 3.39 | 32.01 ± 3.08 | 2.01 ± 2.72 | 0.56, 3.46 | 1.29 ± 1.88 | 0.29, 2.29 |
| Fibrinogen (mg/dL) | |  |  |  |  |  |  |  |
| CKD | FC-low (n=21) ^a^ | 324.5 ± 75.1 | 327.5 ± 61.9 | 307.7 ± 68.3 | 13.8 ± 48.9 | −9.1, 36.6 | −16.8 ± 61.9 | −45.0, 11.4 |
|  | FC-high (n=21) | 329.4 ± 94.2 | 331.8 ± 60.7 | 328.0 ± 81.9 | 2.4 ± 69.6 | −29.3, 34.0 | −1.4 ± 91.7 | −43.2, 40.3 |
| Non-CKD | FC-low (n=15) | 285.2 ± 60.4 | 291.7 ± 60.3 | 267.7 ± 41.5 | 6.5 ± 35.4 | −13.1, 26.1 | −17.5 ± 53.0 | −46.9, 11.8 |
|  | FC-high (n=16) | 279.0 ± 47.6 | 291.5 ± 56.5 | 289.2 ± 53.3 | 12.5 ± 39.7 | −8.7, 33.7 | 10.2 ± 36.2 | −9.1, 29.5 |
| ^a^ Week 8, n=20; ^b^ Week 8, n=19 | | | | | | | | |
| CI, confidence interval; SD, standard deviation; CKD, chronic kidney disease; FC-low group, ferric citrate hydrate at 500 mg (approximately 120 mg elemental iron)/day; FC-high group, ferric citrate hydrate at 1000 mg (approximately 240 mg elemental iron)/day; EOT, end of treatment; PT-INR, prothrombin time-international normalized ratio; APTT, activated partial thromboplastin time | | | | | | | | |
